# Friction and Wear in Nanoscratching of Single Crystals: Effect of Adhesion and Plasticity

**DOI:** 10.3390/nano12234191

**Published:** 2022-11-25

**Authors:** Jianqiao Hu, Qinglei Zeng

**Affiliations:** 1 State Key Laboratory of Nonlinear Mechanics, Institute of Mechanics, Chinese Academy of Sciences, Beijing 100190, China; 2 School of Engineering Science, University of Chinese Academy of Sciences, Beijing 100049, China; 3 Institute of Advanced Structure Technology, Beijing Institute of Technology, Beijing 100081, China

**Keywords:** adhesion and plasticity, friction coefficient, wear rate, molecular dynamics, nanoscratch simulation

## Abstract

Friction and wear are two main tribological behaviors that are quite different for contact surfaces of distinct properties. Conventional studies generally focus on a specific material (e.g., copper or iron) such that the tribological result is not applicable to the other contact systems. In this paper, using a group of virtual materials characterized by coarse-grained potentials, we studied the effect of interfacial adhesion and material plasticity on friction and wear by scratching a rigid tip over an atomic smooth surface. Due to the combined effects of adhesion and plasticity on the nanoscratch process, the following findings are revealed: (1) For shallow contact where interfacial adhesion dominates friction, both friction coefficient and wear rate increase as the adhesion increases to a critical value. For deep contact where plasticity prevails, the variation of friction coefficient and wear rate is limited as the adhesion varies. (2) For weak and strong interfacial adhesions, the friction coefficient exhibits different dependence on the scratch depth, whereas the wear rate becomes higher as the scratch depth increases. (3) As the material hardness increases, both the friction coefficient and wear rate decrease in shallow and deep contacts.

## 1. Introduction

Friction and wear are two main tribological processes due to the relative sliding between contact surfaces and widely exist in mechanical transmission devices. For the two tribological processes, the friction coefficient and specific wear rate are used to characterize the friction and wear, respectively. According to the classical theory of Bowden and Tabor [1], the friction coefficient for contact surfaces can be expressed as the ratio between the interfacial shear strength and the hardness of the softer material in contact. In the empirical wear relation developed by Archard [2], the wear volume per unit sliding distance is proportional to the normal force and the material hardness. Tribological experiments [3,4,5] showed that the friction and wear properties vary greatly in different material systems. By performing the ball-on-plate test for copper of three structures, i.e., the coarse-grained, nano-grained, and gradient nano-grained structures, Chen et al. [6] found that the friction coefficient and wear rate are highest in the coarse-grained copper. The variation of friction and wear properties was explained by the change in material hardness and deformation mechanism in copper with distinct nanostructures. Besides the mechanical properties, external loading conditions also affect the friction and wear behaviors [7,8]. For instance, Chowdhury et al. [9] studied the effect of normal load and sliding velocity on the tribological properties of aluminum using a pin-on-disc test; the results showed that the friction coefficient decreases when the normal load or the velocity increases, whereas the wear rate exhibits an opposite dependence on the normal load and sliding velocity. More experiments were performed to study the friction and wear under different conditions, including incidence angle [10], crystal orientation [11,12], coatings or oxidation [13,14,15], and surface texture [16,17]. In addition, the tribochemical reactions [18] at the sliding interface may also affect the material properties such as interfacial adhesion and material plasticity, resulting in the variation of frictional and wear processes. It can be seen that understanding the friction and wear is complex because both material properties and loading conditions can affect these two tribological processes.

Numerical simulation was widely used to understand the friction and wear process [19,20,21]. For instance, finite element method (FEM) simulations were performed to study the effect of interfacial shear strength and contact interference on the friction coefficient in the collision of two hemispherical asperities [22]. Discrete dislocation dynamics (DDD) was carried out to study the size effect of friction stress in the contact and sliding of asperities [23]; the results provided an insight into the dislocation-dominated plasticity at the microscopic scale. However, for contact surfaces subjected to wear, severe plasticity and even fracture may occur. It is challenging to capture the irreversible damage that occurs during the wear process using the above two numerical methods. Originating from describing interactions between atoms, molecular dynamics (MD) simulations can effectively capture the fracture process without artificial criteria in the tribological system. In the pioneering work of Aghababaei et al. [24], two main adhesive wear mechanisms (i.e., the plasticity-induced asperity smoothing and the fracture-induced wear debris) were demonstrated, where a critical length scale is proposed for the transition of wear mechanism. This transition can be inherently attributed to the material’s brittle/ductile property [25]. More recently, Zhao et al. [26,27] clarified the effect of interfacial adhesion on asperity wear by sliding a deformable indenter on a rigid substrate; the results showed that the dislocation glide dominates the friction in the presence of weak adhesion, leading to a sublinear wear relation. These studies are valuable to understand the wear process, and yet the correlation between friction and wear remains unclear. This may be due to the fact that the definition of the friction coefficient is problematic in the configuration of asperity collision; thus, a multiscale framework was proposed to quantify the friction coefficient at the surface level [28]. More importantly, how do friction and wear properties change in different material systems? It is still an open question, and clarifying this question will help gain control of the friction and wear process in engineering applications.

To address this question, using virtual materials characterized by a series of coarse-grained potentials developed recently [24], we studied the effect of the material hardness and interfacial adhesion on the friction and wear properties. Compared with the conventional potentials used in MD simulations, the coarse-grained potentials have the advantage that the material ductile properties can be changed without disturbing the elastic properties. This provides the chance to clarify the effect of material hardness on friction and wear without introducing other mechanical properties. To mimic the pin-on-disc test, we performed MD simulations by scratching a rigid tip over an atomic smooth surface. The paper is organized as follows. The nanoscratch model is presented in Section 2. The simulation results of friction and wear are presented and discussed in Section 3. Finally, Section 4 summarizes the conclusions.

## 2. Methodology and Model Description

**Figure 1:**
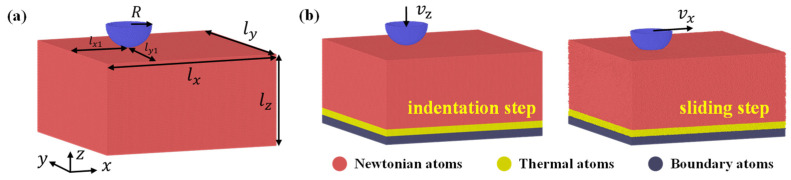
(**a**) Geometry of the nanoscratch model. (**b**) Two-step loading of the scratch process.

The scratch test is performed using the Large-scale Atomic/Molecular Massively Parallel Simulator [29]. As shown in Figure 1, the nanoscratch model consists of a hemispherical tip and a substrate. The atoms in the tip and the substrate are all located in a face-centered cubic (FCC) single crystalline structure. The tip is fabricated by cutting a hemisphere from the FCC crystal, and the tip geometry is similar to the stepped tip [30]. The tip is set to be rigid and has a radius of 15*a* with *a* being the lattice constant. The substrate has a dimension of 120*a* × 120*a* × 60*a* along *x*-[100], *y*-[010] and *z*-[001] crystal orientations; the size of this substrate is large enough for defects evolution during scratching. We also confirmed that a larger box size would not alter the main friction behaviors in this study (see Supplementary Materials). In addition, to avoid the defects moving across the lateral boundaries, the initial position of the tip is located at *l_x_*_1_ = 40*a* and *l_y_*_1_ = 60*a*. The above parameters are also summarized in Table 1.

In the scratch model, the tip is rigid such that the interactions among the tip atoms are ignored. The interactions among atoms in the substrate are described by the coarse-grained potentials [24], and the material ductility can be modified by changing the potential tail. In detail, the coarse-grained potentials are developed based on the modified Morse potentials as follows:

(1) $$\frac{E(r)}{\varepsilon_{0}}=\{\begin{array}{cc} e^{-2\alpha_{0}\left(r-r_{0}\right)}-2e^{-\alpha_{0}\left(r-r_{0}\right)} & r<1.1r_{0}\\ a_{3}r^{3}+a_{2}r^{2}+a_{1}r^{1}+a_{0} & 1.1r_{0}\leq r<r_{c}\\ 0 & r_{c}\leq r \end{array}.$$

Here, E(r) is the potential energy between two atoms and distance r. $\varepsilon_{0}$ is the depth of the potential well and $r_{0}$ is the equilibrium bond distance. $\alpha_{0}$ governs the bond stiffness and is equal to $7.3r_{0}^{-1}$. The truncation at $1.1r_{0}$ ensures the elastic properties are unchanged up to 10% strain. $r_{c}$ is a parameter that governs the tail of interatomic potentials. The parameters $a_{0}\sim a_{3}$ ensure the continuity of the bond energy and the force.

Using this method, we built up four materials of different hardness (details can be found in Appendix A). The potential used between the tip and the substrate, if not explicitly stated, is the same as the one used within the substrate. This indicates that the interfacial strength is the same as the bulk strength. Furthermore, we changed the cohesive energy between the tip and substrate to study the effect of interfacial adhesion.

In the simulations, periodic boundary conditions are imposed along *x* and *y* directions, and the *z* direction is non-periodic. Along the *z* direction, there are three kinds of atoms in the substrate as shown in Figure 1. The boundary atoms at the bottom with a thickness of 5*a* are kept fixed in space. The adjacent atoms of thickness 5*a* are thermostat atoms whose temperature is controlled by the Langevin thermostat method [31]. The other atoms in the substrate are Newtonian atoms obeying the classical Newton’s second law. As shown in Figure 1b, the nanoscratch test is performed with a two-step loading: the indentation step and the sliding step. During the indentation step, the rigid tip moves towards the substrate along *z* axis at a constant velocity of 0.05*r*_0_/*t*_0_ (*r*_0_ is a reduced length unit and corresponds to the equilibrium bond length between atoms; *t*_0_ is the reduced time unit). After the tip approaches the desired depth, the system is further relaxed for 100*t*_0_ to ensure a relatively stable contact force. Subsequently, the sliding step is imposed by applying a constant tangential velocity of 0.05*r*_0_/*t*_0_ on the rigid tip along the *x* direction with a sliding distance of 60*r*_0_. During the sliding step, the tangential force *F_τ_* along *x* direction and the normal force *F_n_* in *z* direction is calculated every 0.1*r*_0_ in the simulations.

During the sliding step, the interaction forces between the tip and substrate are calculated. Based on the interaction forces, two methods were used to determine the friction coefficient [32]. First, the friction coefficient is obtained from the slope of the friction force as a function of normal load. This approach eliminates the contribution from adhesive force and determines the friction coefficient at a macroscopic concept. It was used in the nanoscratching of Al [33], and the friction coefficient was calculated by linear fitting the tangential and normal forces at different scratch depths. Second, based on a single-point measurement, the friction coefficient was defined by directly dividing the tangential force by the normal force. This method was widely used in the single-asperity contact, e.g., the nanoscratch simulations [34] and the microscratch test [35]. In this work, we studied the effect of scratch depth on the friction coefficient and thus used the second method to calculate the friction coefficient at each scratch depth based on the single-point measurement. During the nanoscratching, the instantaneous friction coefficient is calculated as the ratio of the tangential force *F_τ_* and the normal force *F_n_*. For each scratch depth, we followed the method used in [36] to determine the friction coefficient which is calculated by averaging the instantaneous friction coefficient. In this study, the friction coefficient is averaged over the last sliding distance of 30*r*_0_, and the standard deviation of the instantaneous friction coefficient is calculated to present the error bar of the friction coefficient. In addition to the instantaneous forces, we also calculated the averaged tangential force ${\bar{F}}_{\tau }$ and the averaged normal force ${\bar{F}}_{n}$ over the last distance of 30*r*_0_ to analyze the contact force during friction.

For nanoscratching on an atomistic flat surface, worn atoms can be evaluated as the atoms located above the original substrate surface [37]. Herein, we adopted a threshold height [38] to determine the worn atoms. If the location of an atom is above the substrate surface with a threshold height of 1.0*r*_0_, the atom is taken as a worn atom. Once the number of worn atoms is obtained, the wear volume can be calculated by multiplying the number and the volume of an atom arranged in a perfect crystal. Then, according to the Archard wear law [2], we can obtain the specific wear rate *k_a_* as

(2) $$k_{a}=\frac{V_{wear}}{F_{n}\times S}\;,$$

where *V_wear_* is the accumulated wear volume corresponding to the sliding distance *S*.

In the analysis, the dislocation extraction algorithm (DXA) [39] method is performed to track dislocations, and the open-source software OVITO [40] is used to visualize the defect structures.

## 3. Results and Analysis

### 3.1. Friction and Wear during Scratching

**Figure 2:**
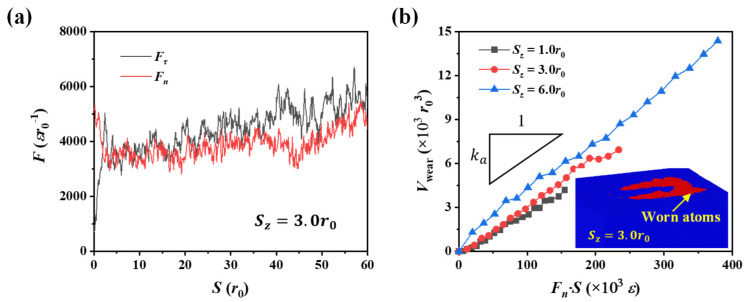
(**a**) Tangential and normal forces between the tip and substrate at a scratch depth of 3.0*r*_0_. (**b**) Wear volume as a function of the product of the normal force and sliding distance; the inset shows the worn atoms at a depth of 3.0*r*_0_ at the sliding distance of 60*r*_0_. The scratch test is performed in material of *r_c_* = 1.22*r*_0_.

In this section, we studied the friction and wear in relatively brittle material (*r_c_* = 1.22*r*_0_). Figure 2a shows the force-displacement at a scratch depth of *S_z_* = 3.0*r*_0_ during the sliding step. It can be seen that the tangential force and normal force become relatively stable after the initial increase. The fluctuations in the interaction forces during scratching can be attributed to the formation and movement of dislocations beneath the tip [41]. Due to the force fluctuations, we averaged the instantaneous friction coefficient within the sliding distance ranging from 30*r*_0_ to 60*r*_0_ to quantify the friction coefficient for each scratch depth. Furthermore, to demonstrate the reliability of simulation results, we performed another two sets of nanoscratch simulations; it was confirmed that there is little difference in the concerned results over a wide range of scratch depths (see Supplementary Materials).

Figure 2b presents the evolution of wear volume as the scratching continues, and the inset illustrates the worn atoms (in red color) when the scratch depth is 3.0*r*_0_ and the sliding distance is 60*r*_0_. It can be seen that the accumulated wear volume is proportional to the product of the normal force and sliding distance, which follows the Archard wear law. By linearly fitting the relationship in Figure 2b, we can obtain the wear rate *k_a_* in Equation (2), and the error bars of *k_a_* correspond to 95% confidence intervals for the fitted values.

### 3.2. Effect of Interfacial Adhesion

For contact surfaces in engineering applications, the interfacial adhesion varies due to different contact materials [42], contact geometry [30], and even tribochemical reactions [18]. To clarify the role of interfacial adhesion during friction, we changed the cohesive energy in the Morse potential between the tip and substrate. The depth of the potential well $\varepsilon_{0}$ in Equation (1) is changed by $\varepsilon_{adh}$ with the adhesion ratio $\varepsilon_{adh}/\varepsilon_{0}$ ranging from 0.2 to 1.5. In this way, we essentially modified the interfacial strength with the ratio of interfacial strength to bulk strength ranging from 0.2 to 1.5.

#### 3.2.1. Friction Coefficient

**Figure 3:**
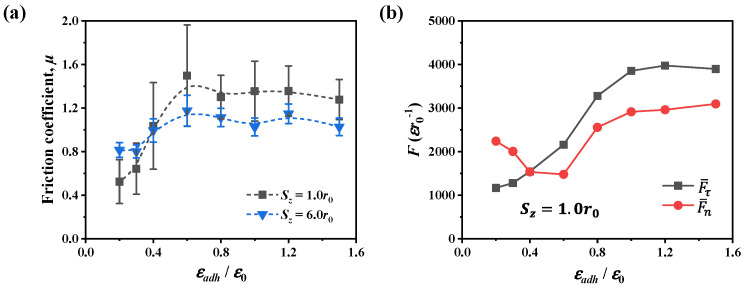
(**a**) Friction coefficient at two scratch depths as the interfacial adhesion changes, and the dashed lines are guides for eyes. (**b**) Averaged tangential and normal forces between tip and substrate at a depth of 1.0*r*_0_. Scratch test is performed in material of *r_c_* = 1.22*r*_0_.

Figure 3a presents the variation of the friction coefficient over a wide range of adhesion ratios from 0.2 to 1.5 in both shallow and deep contacts. For shallow contact of *S_z_* = 1.0*r*_0_, the friction coefficient first increases and then remains relatively stable as the adhesion increases, and the critical adhesion ratio for the transition is ~0.6. We first studied the adhesion effect in shallow contact by analyzing the interaction forces. As shown in Figure 3b, the averaged tangential force ${\bar{F}}_{\tau }$ and the averaged normal force ${\bar{F}}_{n}$ are presented. It can be seen that when the adhesion ratio exceeds ~0.6, both the tangential force and normal force vary similarly; this indicates that in this case the interfacial strength is strong enough to transfer the normal force to the shear force during scratching. As a result, the friction coefficient exhibits a weak dependence on the adhesion. In contrast, when the adhesion ratio is below ~0.6, although the tangential force remains increasing as the adhesion increases, the normal force decreases as the adhesion becomes stronger. This variation of interaction force is consistent with the scratch results in [43] where the normal force decreases when the adhesion between C and Fe (the tip and substrate) increases. Particularly, when the adhesion ratio is larger than 1.0, the tangential force and normal force remain relatively stable. This is because the interfacial strength is larger than the bulk strength in this situation, such that the bulk strength dominates the scratch process.

**Figure 4:**
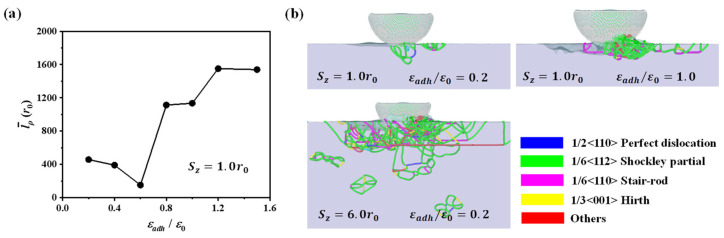
(**a**) Averaged dislocation length as the adhesion increases at the scratch depth of 1.0*r*_0_. (**b**) Dislocation structures at the scratch distance of 30*r*_0_ for two adhesion ratios; only a part of the substrate is shown.

For deep contact of *S_z_* = 6.0*r*_0_, the variation of the friction coefficient is smaller at different adhesion levels when compared with the case of shallow contact. During sliding, both interfacial adhesion and contact plasticity [44,45] can affect the interactions between contact surfaces. In deep contact, the adhesion effect is limited due to the increase of dislocation plasticity. Therefore, to further interpret the adhesion effect in shallow and deep contacts, we analyzed the dislocation plasticity during scratching as shown in Figure 4. In the analysis, we first tracked the dislocations at each timestep using the dislocation extraction algorithm (DXA) [39]. Then the dislocation length was calculated by summing all dislocations in the substrate. Based on the above analysis, we averaged the dislocation length within the sliding distance ranging from 30*r*_0_ to 60*r*_0_ to quantify the dislocation plasticity. As the adhesion ratio increases, the change of dislocation length/density shown in Figure 4a is similar to the variation of the normal force in Figure 3b. Corresponding to the lowest normal force at the adhesion ratio of 0.6, the dislocation density is the smallest. When the adhesion ratio exceeds ~0.6, the number of dislocations becomes larger, and the normal force increases as the adhesion becomes stronger. The increase of dislocation density results in higher tangential and normal forces, which may indicate that the localized strain hardening occurs as a result of numerous dislocations. In contrast to the case of strong adhesion, the dislocation density is smaller when the adhesion ratio is below ~0.6. In this situation, the interfacial strength is not strong enough to transfer the shear loading such that the dislocation plasticity is limited, and then the interfacial adhesion is the controlling factor for friction. From the above analysis, at the shallow depth of 1.0*r*_0_, there exists a critical adhesion ratio of ~0.6 that the dominant friction factor changes from interfacial adhesion to dislocation plasticity.

Figure 4b illustrates the dislocation structures at two adhesion levels. In shallow contact, the interfacial adhesion dominates the scratch process such that more dislocations can be observed beneath the tip as the adhesion ratio increases from 0.2 to 1.0. In this case, the stronger adhesion between the tip and substrate results in a higher tangential force during scratching, and thus more dislocations are generated beneath the tip. Furthermore, at the adhesion ratio of 0.2, the number of dislocations increases dramatically as the scratch depth increases from 1.0*r*_0_ to 6.0*r*_0_. The dominant friction factor changes from the interfacial adhesion to the dislocation plasticity. In deep contact, there exist a large number of dislocations, and contact yielding occurs. Then, the friction coefficient can be roughly evaluated by the ratio between shear strength and contact hardness and thus remains relatively stable over a wide range of adhesion levels as shown in Figure 3a.

**Figure 5:**
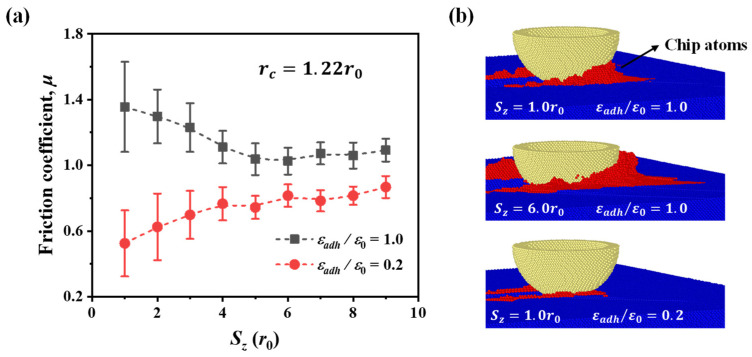
(**a**) Friction coefficient for two adhesion ratios at different scratch depths. (**b**) Atomic configuration at the scratch distance of 45*r*_0_.

In Figure 3a, an interesting feature can be observed in the evolution of the friction coefficient. For weak adhesion (e.g., $\varepsilon_{adh}/\varepsilon_{0}$ = 0.2), the friction coefficient becomes larger as the scratch depth increases from 1.0*r*_0_ to 6.0*r*_0_. However, for the case of strong adhesion, the friction coefficient exhibits opposite depth dependence and decreases at a deeper scratch depth. This feature can be further confirmed in Figure 5a. For strong adhesion with $\varepsilon_{adh}/\varepsilon_{0}$ = 1.0, the friction coefficient is high in shallow contact and decreases to relatively stable as the scratch depth increases to ~5.0*r*_0_. The high friction coefficient in shallow contact may be induced by numerous chip atoms accumulating in front of the tip due to the strong adhesion, as shown in Figure 5b. The chip atoms can be considered as a part of the worn atoms in front of the tip in this study, and the formation of chip atoms was widely observed during nanoscratching [46,47]. These chip atoms greatly contribute to the tangential resistance and thus increase the friction coefficient. Note that although there are also numerous chip atoms in deep contact, the effect of chip atoms on the friction coefficient is limited compared with shallow contact. This is because the normal force is much smaller in shallow contact and thus the same increase in tangential resistance leads to a more significant increase in friction coefficient. For weak adhesion with $\varepsilon_{adh}/\varepsilon_{0}$ = 0.2, the friction coefficient varies differently from the case of strong adhesion. The friction coefficient first increases and then remains relatively stable. Such variation of the friction coefficient is consistent with the results in the diamond–iron [48] and diamond–copper [49] scratch systems in that the friction coefficient is low in shallow contact and becomes higher as the scratch depth increases. Particularly, in the study of the diamond–copper scratch system [49], it was also demonstrated that the effect of interfacial adhesion at deep scratching depth is limited. The experimental observation in the shale rock–dry quartz system [50] demonstrated the same variation of the friction coefficient as well.

#### 3.2.2. Wear Rate

**Figure 6:**
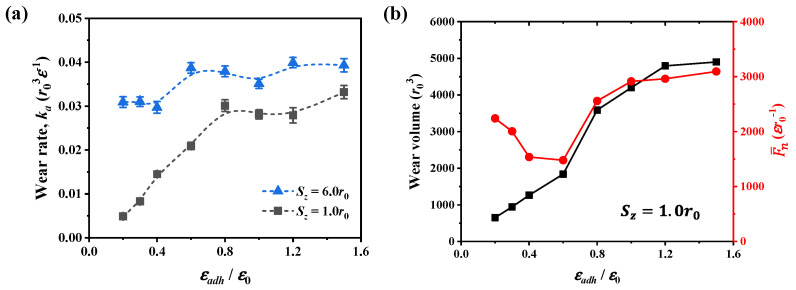
(**a**) Wear rate at two scratch depths as the interfacial adhesion changes, and the dashed lines are guides for eyes. (**b**) Wear volume and averaged normal force at the depth of 1.0*r*_0_. The wear volume is obtained at the scratch distance of 60*r*_0_.

Similar to the analysis of the friction coefficient, we calculated the wear rate at adhesion ratios ranging from 0.2 to 1.5 for two scratch depths, as shown in Figure 6a. For deep contact of *S_z_* = 6.0*r*_0_ where dislocation plasticity dominates the friction, the wear rate does not show obvious dependence on the adhesion. In contrast, at the depth of 1.0*r*_0_, the interfacial adhesion has a more significant effect on the wear rate, characterized by the more apparent variation of the wear rate at different adhesion levels. In shallow contact, the wear rate exhibits a similar dependence on the interfacial adhesion as the friction coefficient. As the adhesion increases, the wear rate first increases and then remains relatively stable. Correspondingly, the wear volume and normal force are shown in Figure 6b. The wear volume first increases and then remains relatively stable as the adhesion increases, similar to the evolution of tangential force in Figure 3b. This variation of wear volume is consistent with the material removal observed in Tungsten [51] in that the number of removal atoms first increases and then remains stable as the adhesive strength becomes stronger. In contrast, compared with the wear volume, the normal force exhibits a reversed dependence on the adhesion when the adhesion ratio is below ~0.6, as analyzed in Figure 3b.

**Figure 7:**
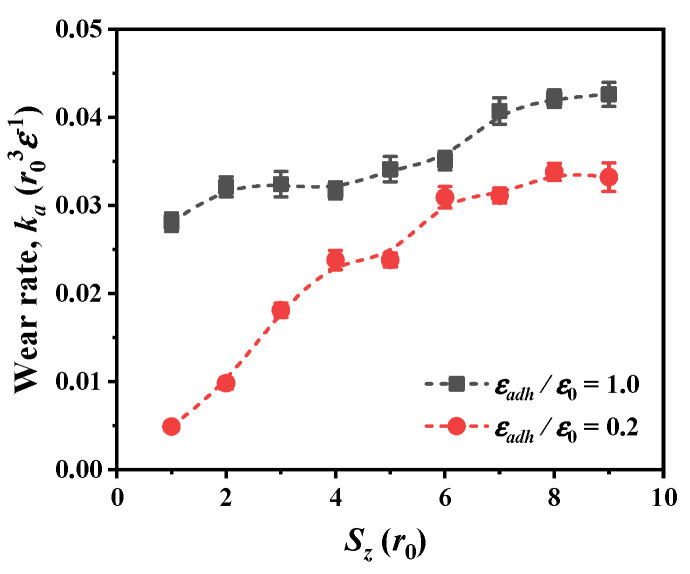
Wear rate for two adhesion ratios at different scratch depths in material of *r_c_* = 1.22*r*_0_.

During the scratching, the wear rate increases with increasing scratch depth at both weak and strong adhesions, and the variation of wear rate is smaller at strong adhesion, as shown in Figure 7. Also, as the adhesion ratio increases from 0.2 to 1.0, and the wear rate becomes higher for all scratch depths. The variation of wear rate can be explained by analyzing the dislocation plasticity in Figure 4b and the formation of chip atoms in Figure 5b. For scratching with strong adhesion, more dislocation plasticity would be produced because the stronger adhesion results in a higher shear loading during scratching. The increase of dislocation plasticity produces more worn atoms, resulting in a higher wear rate.

From the above analysis, it can be seen that the interfacial adhesion dominates the friction and wear when the scratch depth is small and the adhesion strength is below a critical value. In this case, the friction coefficient and wear rate vary significantly as the adhesion changes. While for deep contact and strong adhesion, the friction coefficient and wear rate remain relatively stable. The strong interfacial strength can fully transfer the normal force to the tangential force, and a large number of dislocations are generated during scratching. Therefore, plasticity dominates the friction and wear in this situation.

### 3.3. Friction and Wear in Materials of Different Ductility

**Figure A1:**
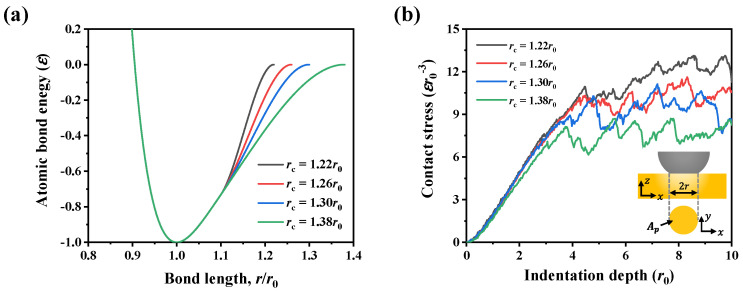
(**a**) The bond energy versus atomic bond length for different cut-off radii *r_c_*. (**b**) Contact stress for materials with potentials of different *r_c_*; the inset illustrates the indentation model with an indenter of radius 15*a*.

Many of the conventional studies focus on friction and wear in specific material systems, e.g., the zirconia composites [52], the magnesium alloy [53], and the molybdenum-based systems [54]. However, due to the differences in material properties, a deep understanding of friction behavior remains ambiguous. Here, inspired by the work of Aghababaei et al. [24], we constructed materials of different plasticity/hardness using the modified Morse potentials. As shown in Table A1 and Figure A1a given in Appendix A, a smaller cut-off radius *r_c_* results in a larger hardness and corresponds to the more brittle material. With this group of potentials, we performed the scratch test to understand the friction and wear in materials of different ductility. In the simulation, the interfacial strength is set to the same as the bulk strength, i.e., the adhesion ratio is 1.0.

**Figure 8:**
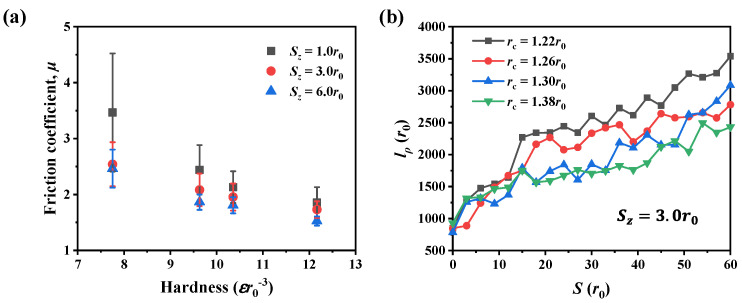
(**a**) Frictional coefficient in materials of different hardness. (**b**) Evolution of dislocation length in four materials as the scratching advances.

Figure 8a presents the variation of the friction coefficient in materials of different hardness. Results showed that the friction coefficient decreases as the hardness becomes larger for the three scratch depths. Corresponding to the increase of hardness (the decrease of the cut-off radius *r_c_*), the number of dislocations also increases, as shown in Figure 8b. Note that these materials with different hardness have the same elastic properties, including Young’s modulus and Poisson’s ratio. Therefore, the variation of hardness can be mainly attributed to the change of dislocation plasticity. In addition, it can be seen that for the material of specific hardness, the friction coefficient decreases as the scratch depth increases. This variation is because of the strong adhesion and was analyzed in Section 3.2.

**Figure 9:**
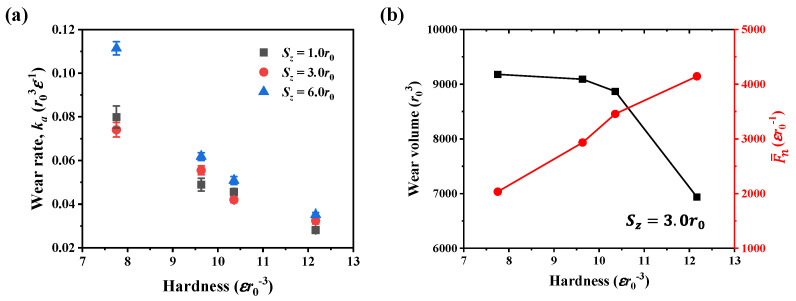
(**a**) Wear rate in materials of different hardness. (**b**) Wear volume and averaged normal force at the scratch depth of 3.0*r*_0_.

Furthermore, the wear rate for materials of different hardness is shown in Figure 9a. It can be seen the wear rate exhibits the same hardness dependence as the friction coefficient; that is, as the material hardness increases, the wear rate decreases. The hardness dependence of the wear rate can be explained by the variation of wear volume and normal force, as shown in Figure 9b. At the scratch depth of 3.0*r*_0_, for ductile material, the increase of wear rate is primarily due to the increase in normal force because the variation of wear volume is limited as the hardness increases. In contrast, for the brittle material with hardness of 12.2$\varepsilon r_{0}^{-3}$, the wear rate decreases not only because of the increase in normal force but also the reduction of wear volume.

## 4. Summary and Conclusions

In this study, using a group of materials characterized by modified Morse potentials, we studied the effect of adhesion and plasticity on the friction and wear properties using a nanoscratch test in single crystals. Due to the variation of dominated deformation mechanisms, distinct friction and wear phenomena are observed. Our findings may provide some ideas for the design and regulation of tribology by modifying the interfacial adhesion and material properties in tribological systems. The main findings are summarized as follows:

(1) For shallow contact where the interfacial adhesion dominates the friction, there exists a critical adhesion ratio of ~0.6, below which the friction coefficient and wear rate increase as the adhesion increases, and above which the friction coefficient and wear rate remain relatively stable. For deep contact where dislocation plasticity prevails, the variation of the friction coefficient and wear rate is limited as the adhesion increases.
(2) The friction coefficient exhibits different dependence on the scratch depth in situations with different adhesion. For strong adhesion, the friction coefficient is high in shallow contact and decreases at a deeper scratch depth, whereas for weak adhesion, the friction coefficient is lower in shallow contact. Meanwhile, the wear rate becomes higher as the scratch depth increases for both weak and strong adhesions.
(3) As the material hardness increases, both the friction coefficient and wear rate decrease. For ductile materials, the normal force primarily affects the wear rate, and the variation of wear volume is limited. For relatively brittle materials, the increase of normal force and the reduction of wear volume lead to the decreased wear rate as the hardness increases.

## Figures and Tables

**Table 1:** Parameters for the scratch test with coarse-grained potentials.

Parameters	Values/Expression
Tip radius	*R* = 15*a*, *a* = $\sqrt{2}r_{0}$
Substrate size	*l_x_* = 120*a*, *l_y_* = 120*a*, *l_z_* = 60*a*
Initial position of tip	*l_x_*_1_ = 40*a*, *l_y_*_1_ = 60*a*
Time step (*t*_0_)	0.002
Temperature (*ε*/*k*_B_)	0.1
Scratch velocity (*r*_0_/*t*_0_)	0.05
Scratch depth (*r*_0_)	1.0~9.0
Scratch direction	[100] on (001) surface

## Data Availability

The data that support the findings of this study are available from the corresponding author upon reasonable request.

## Supplementary Materials

The following are available online at https://www.mdpi.com/article/10.3390/nano12234191/s1, Figure S1: Evaluation of simulation box size: (a) the tangential forces for three models, and (b) the friction coefficient at three scratch depths; Figure S2: (a) Tangential force and normal force at the depth of 3.0*r*_0_ in three samples, and (b) the friction coefficient at different scratch depths. References [33,55,56] were cited in Supplementary Materials.

## Appendix A

This appendix introduces the details of the coarse-grained interatomic potentials. As listed in Table A1 and shown in Figure A1a, we constructed four model materials in this study by adopting different cut-off radii *r_c_* in the modified potentials. The potential with a shorter cut-off radius (*r_c_* = 1.22*r*_0_) corresponds to a more brittle material. In contrast, the increase in the cut-off radius (e.g., *r_c_* = 1.38*r*_0_) makes the material more ductile. Therefore, using these model potentials, we essentially studied the friction and wear law in different material systems. All the models with modified Morse potentials are constructed in the FCC structure with the lattice constant *a* being $\sqrt{2}r_{0}$. The Young’s modulus for all materials is 150$\varepsilon r_{0}^{-3}$ [25]. The indentation is carried out at the temperature of 0.1*ε/k*_B_ and the velocity of 0.01*r*_0_/*t*_0_, and the results are shown in Table A1 and Figure A1b. The material hardness is calculated by averaging the contact stresses at depths between 6*r*_0_ to 10*r*_0_. It can be seen that a more brittle material has a larger hardness in this model.

**Table A1:** Materials with modified Morse potentials and the hardness.

Cut-Off Radius, $r_{c}$ $\;(r_{0})$	Potential Well (ε)	Young’s Modulus $\;(\varepsilon r_{0}^{-3})$	Hardness $\;(\varepsilon r_{0}^{-3})$
1.22	1.0	150	12.2
1.26	1.0	150	10.4
1.30	1.0	150	9.6
1.38	1.0	150	7.8
