# Supplementary material for: Friction and Wear in Nanoscratching of Single Crystals: Effect of Adhesion and Plasticity
Source: Nanomaterials (Basel). 2022 Nov 25;12(23):4191. doi: 10.3390/nano12234191 (PMC9741148; doi:10.3390/nano12234191)
Supplement: Supplementary file 1 [file nanomaterials-12-04191-s001.zip › nanomaterials-2022975-supplementary.pdf]

Supplementary Materials

1. Evaluation of simulation box size

In the study, the substrate has a dimension of $120a \times 120a \times 60a$ along x-[100], y-[010], and z-[001] crystal orientations, and a is the lattice constant. In the open literature, we found that many models are as large as or smaller than our model. For example, in the nanoscratch simulation of Fe [1], the box size is $100a \times 100a \times 50a$; and for Cu [2], the box size is $70a \times 50a \times 33a$. These studies can obtain the main features of friction and wear in the related materials. Particularly, in the nanoscratching of Al [3], the simulation results showed that the friction coefficient becomes relatively stable as the thickness is larger than ~$16a$.

Furthermore, we also discussed two other larger model sizes, i.e., $180a \times 120a \times 60a$ and $120a \times 120a \times 80a$. As shown in Figure S1, a larger box would not alter the main friction behaviors in this study. Thus, the model size used in this study is considered large enough.

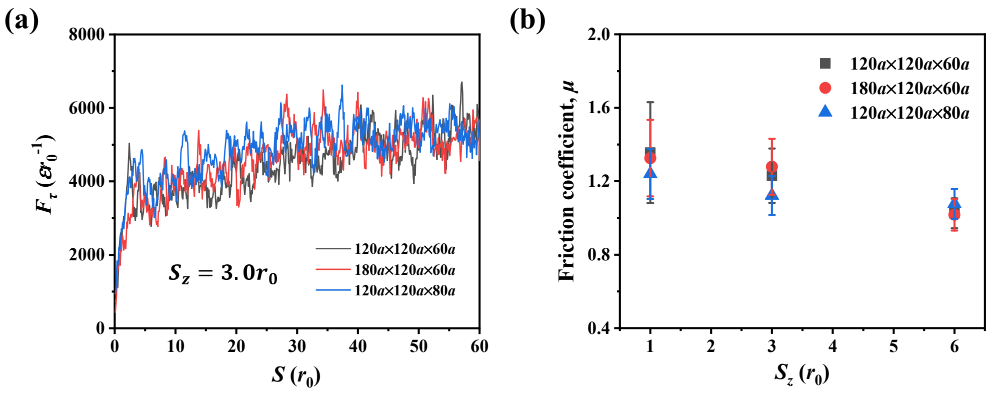

Figure S1. Evaluation of simulation box size: (a) the tangential forces for three models, and (b) the friction coefficient at three scratch depths.

2. Reliability of simulation results

To demonstrate the reliability of simulation results, we performed another two sets of nanoscratch simulations. As shown Figure S2, it was confirmed that there is little difference in the concerned results over a wide range of scratch depths.

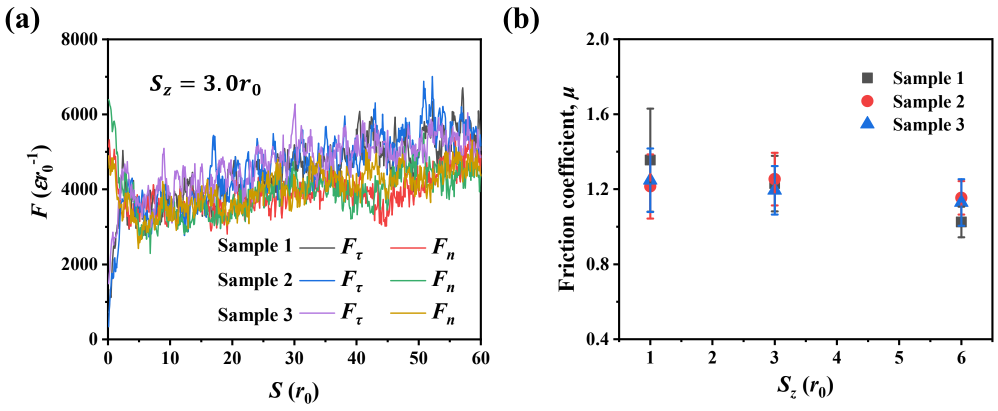

Figure S2. (a) Tangential force and normal force at the depth of $3.0r_0$ in three samples, and (b) the friction coefficient at different scratch depths.

References

[1] Y. Gao, H.M. Urbassek, Scratching of nanocrystalline metals: A molecular dynamics study of Fe. *Applied Surface Science* **2016**, *389*, 688-695.

[2] J. Li, Q. Fang, Y. Liu, L. Zhang, Scratching of copper with rough surfaces conducted by diamond tip simulated using molecular dynamics. *The International Journal of Advanced Manufacturing Technology* **2014**, *77*, 1057-1070.

[3] T. Junge, J.-F. Molinari, Plastic activity in nanoscratch molecular dynamics simulations of pure aluminium. *International Journal of Plasticity* **2014**, *53*, 90-106.
